# Caregiver knowledge and Attitudes toward Human Papillomavirus Vaccination among adolescent girls living with HIV in Sierra Leone: A health facility-based cross-sectional study

**DOI:** 10.64898/2026.09.21.26363609

**Published:** 2026-09-23

**Authors:** Darlinda F Jiba, Mamadu Baldeh, Patrick Turay, Saidu Kanu, Matilda N Kamara, Lynda M. L Farma, Umu Barrie, Waheed O Awonuga, Mary M Baio, Daniel Sesay, Diana Shehab, Rosaline Sinnah, Enanga S Namanga, Phildys M D Johnson, Sulaiman Lakoh, J. Andrew Dykens

## Abstract

**Objective:**

To assess the knowledge, willingness and barriers to human papillomavirus (HPV) vaccination among caregivers of adolescent girls living with HIV in Sierra Leone.

**Methods:**

This cross-sectional study examined HPV vaccine knowledge, willingness, and barriers among 249 caregivers of adolescent girls living with HIV in Sierra Leone. Data were collected through structured questionnaires at public tertiary hospitals and high-volume HIV clinics, and associations were assessed using chi-square tests, logistic regression, and Kruskal-Wallis analyses.

**Results:**

Only 32.1% (80/249) of caregivers had heard of HPV, with even fewer aware of its link to cervical cancer (24.4%, 60/246) or prevention methods of cervical cancer (31.9%, 79/248). Misconceptions were prevalent; 16.3% (36/221) erroneously linked oral contraceptives to cervical cancer, and 61.1% (44/72) of caregivers unwilling to vaccinate cited distrust in vaccine efficacy. While tertiary-educated caregivers exhibited the highest level of knowledge (45.5%, p < 0.001), their willingness to vaccinate (50%, 33/66) was comparable to that of less-educated groups, highlighting a knowledge-action gap. Structural barriers dominated, with 66.1% (154/233) citing unawareness of the vaccine and 10.3% (24/233) missing school-based vaccination days. Relationship dynamics significantly influenced willingness: extended family members showed the highest willingness (69.4%, p = 0.029), whereas grandparents were least willing (31%, 9/29). Although systemic gaps such as disrupted vaccination services, vaccine supplies and cold chain limited uptake, healthcare workers emerged as the most effective information source (rank sum = 889.5, p = 0.039).

**Conclusion:**

The study highlights the need for culturally tailored education to dispel myths and community-driven strategies engaging trusted actors like healthcare workers and extended families. This study justifies the need for integration of HPV vaccination and cervical cancer screening into HIV care services.

## Full Text Availability

The license terms selected by the author(s) for this preprint version do not permit archiving in PMC. The full text is available from the preprint server.

